# Bivalirudin for cardiopulmonary bypass in a patient with heparin allergy

**DOI:** 10.1186/s13019-023-02359-2

**Published:** 2023-09-11

**Authors:** Emre Boysan, Renda Circi, Osman Fehmi Beyazal, Erol Şener

**Affiliations:** 1 Department of Cardiovascular Surgery, Medicana International Ankara Hospital, Ankara, Turkey; 2 Department of Cardiovascular Surgery, Başakşehir Çam and Sakura City Hospital, İstanbul, Turkey; 3 Cardiovascular Surgery Department, Başakşehir G-434 Street No: 2L, 34480 Başakşehir, İstanbul Turkey

**Keywords:** Bivalirudin, Heparin allergy, Cardiopulmonary bypass

## Abstract

**Background:**

Hypersensitivity reactions to heparin are uncommon conditions but pose a serious clinical problem for patients requiring cardiopulmonary bypass. Bivalirudin is a reversible direct thrombin inhibitor that can be used instead of heparin.

**Case Report:**

A 49-year-old male patient was admitted to our hospital for coronary artery bypass graft operation with mitral insufficiency and tricuspid valve insufficiency. Heparin allergy was confirmed by skin biopsy and skin tests. Due to this allergy, we used bivalirudin (Bivacard VEM drug, Turkey) during the surgery. A loading dose of 1.0 mg/kg (100 mg) bivalirudin was administered through the central line and a continuous infusion of 2.5 mg/kg/h of the anticoagulant was initiated following the approved protocol. Serial ACTs were obtained at 15-minute intervals during the procedure and the measurements were 330s, 320s, 350s, 360s, and 340s consecutively. Additional boluses of 0.5 mg/kg (50 mg) were administered for each measurement. Left anterior descending, obtuse marginal arteries and the right coronary artery were grafted with the left internal mammary and saphenous veins. Also, mitral valve replacement with St Jude mechanical heart valve and tricuspid ring annuloplasty was performed with Medtronic Duran ring. After the surgery, the patient had an uneventful period in the postoperative intensive care unit with a total of 600ml and 300ml chest tube drainage for two days and was discharged on the 7th day.

**Conclusion:**

Alternative anticoagulation strategies are needed for cardiopulmonary bypass in patients unable to use heparin. Bivalirudin may be recommended as a viable alternative anticoagulant in patients with heparin allergy during cardiopulmonary bypass. However, each patient should be evaluated individually and it should not be forgotten that more than recommended doses may be needed.

## Introduction

Hypersensitivity reactions to heparin and heparin-like compounds are rare [1]. Management of this rare condition for patients requiring cardiopulmonary bypass (CPB) represents therapeutic challenges. An alternative anticoagulant to heparin is required in the aforementioned patients.

Bivalirudin is a small oligopeptide analog of hirudin acting on thrombin through direct inhibition. It has a half-life of 25 min in the presence of normal renal function and is mostly cleared from the circulation by proteolytic enzymes. The minority of bivalirudin is cleared by the renal system. Bivalirudin level has been shown to correspond to activated clotting time (ACT) [2]. These pharmacological properties enable its effective use during CPB [3].

We report a case of coronary artery bypass surgery, mitral valve replacement and tricuspid ring annuloplasty using bivalirudin for anticoagulation during CPB in a patient with hypersensitivity to heparin.

### Case report

A 49-year- old man was admitted to our hospital for coronary artery bypass graft operation (CABG) with concomitant ischemic mitral insufficiency and moderate tricuspid valve insufficiency. Patient characteristics and laboratory parameters are shown in Table 1. He denied any allergies to medications. His past medical history was significant for coronary artery disease and a history of heavy smoking. We ordered enoxaparin, bemiparin, and unfractionated heparin one by one during the preoperative evaluation period but a generalized erythematous skin rash was noted on her arms and chest shortly after each. Skin biopsy and skin tests were performed and heparin allergy was confirmed. Due to this allergy, we decided to use bivalirudin (Bivacard VEM ilaç, Türkiye) during surgery. According to the study of Koster et al., it has been shown that bivalirudin plasma level has been shown to correspond in an almost linear relationship to ecarine clotting time (ECT) [2]. However ECT was not routinely available in our hospital and we decided to use ACT which had been shown to correlate with bivalirudin concentrations nearly in the same manner as ECT [4]. We used The i-STAT^®^ Celite Activated Clotting Time (CeliteACT) test cartridges and i-STAT^®^1 blood analyzer (Abbot, Princeton, New Jersey, USA) in our clinic.

**Table 1:** Patient characteristics and preoperative laboratory parameters

Height (cm)	163
Weight (kg)	97
BMI (kg/m^2^)	1,61
BSA (m^2^)	21,5
White blood cells (x10^9^/L)	8,2
Red blood cells (x10^12^/L)	5,2
Hemoglobin (g/dL)	13,7
Hematocrit (%)	40,2
Platelet (x10^9^/L)	332
International Normalized Ratio	1,17
Prothrombin time (sec)	13
Activated partial thromboplastin time (sec)	32
Glucose (mg/dL)	97
Urea (mg/dL)	33
Creatinine (mg/dL)	1,11
Glomerular filtration rate (ml/dk/m^2^)	74,8
ASAT (U/L)	31
ALAT (U/L)	33
Total protein (g/L)	6,3
Albumin (g/L)	3,7

A Sorin Inspire oxygenator (Sorin Group Italia, Mirandola, Italy) was set up on a Jostra HL20 heart-lung machine (Jostra AB, Lund, Sweden). The circuit was primed with 1500ml ringer lactate solution, 100ml mannitol 20%, and 50 mg bivalirudin. Non-coated CPB circuits are used and no cell-saver is used. After standard monitoring, a right radial artery catheter and a jugular central venous pressure catheter were inserted and normal saline was used for flushing. Routine intravenous general anesthesia with propofol, fentanyl, and pancuronium bromide was performed. Baseline ACT was measured as 138s. After the harvesting of the mammary artery was completed, a loading dose of 1.0 mg/kg (100 mg) bivalirudin was administered through the central line for the 97 kg patient and a continuous infusion of 2.5 mg/kg/h of the anticoagulant was initiated as in the CHOOSE ON study [3]. ACT was obtained 3 min after loading dose and found 400s. An additional bolus of 0.25 mg/kg (25 mg) was administered for safety issues. Aorto-bicaval cannulation was done and on proceeding to CPB, the bivalirudin infusion was administered through the perfusion circuit at the same rate. The patient’s nasopharyngeal temperature was maintained at 34^0^ C while on CPB.

**Fig. 1:**
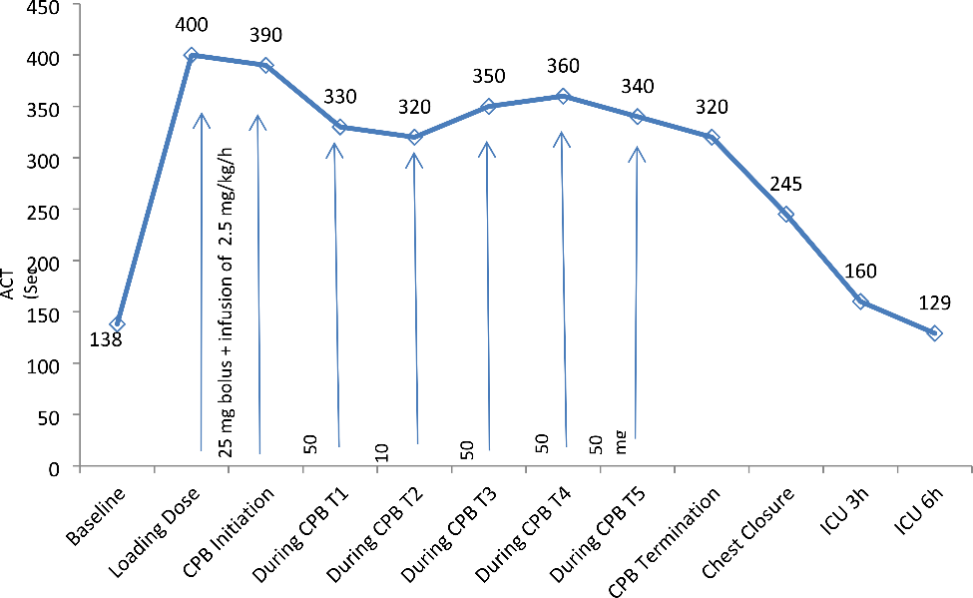
Perioperative monitoring of ACT and doses of bivalirudin. ICU: Intensive care unit, T1:15 min, T2:30 min, T3:45 min, T4:60 min, T5:75 min

After clamping of the aorta, cold blood cardioplegia was administered through the aortic root cannula. We gave close attention to maintaining blood volume in the cardiotomy reservoir below 600ml to avoid possible stagnation. ACT measurement was obtained and ACT was found to be 390s. An additional bolus of 0.5 mg/kg (50 mg) was administered for increasing ACT above 400s which is a generally accepted time for proceeding CPB [4]. The left anterior descending artery, obtuse marginal arteries and right coronary artery were grafted with the left internal mammary artery and great saphenous vein. Also, mitral valve replacement with a #31 St Jude mechanical heart valve and tricuspid ring annuloplasty was performed with #31 Medtronic Duran ring. Serial ACT’s were obtained at 15 min intervals during the CPB and the measurements were 330s, 320s, 350s, 360s, and 340s consecutively (Fig. 1). Additional boluses of 0.5 mg/kg (50 mg) were administered for each measurement. In addition to these, after 320s measurement, we realized that there was a cloudy and creamy image in the reservoir and we administered an extra 1 mg/kg (100 mg) bolus and increased continuous infusion of 2.5 mg/kg/h to 3.5 mg/kg/h to maintain desired ACT (400s). At first we decided to operate in mildly hypothermic conditions, but when it was below our target for ACT, we decided to cool the patient to 30^0^ C. Because proteolytic activity is responsible for the elimination of bivalirudin, we wanted to reduce the proteolytic activity with this extra cooling and increase the infusion rate at the same time [5], Rewarming was started after opening the clamp while performing the proximal anastomosis. We reached 37^0^ C and stopped the infusion and waited 25 min before termination of CPB because bivalirudin has a half-life of about 25 min [2].

After discontinuation of CPB, ACT was measured as 320s. Two packs of fresh frozen plasma and 3 packs of platelet suspension were transfused to the patient. Despite an abnormal ACT (245s) 15 min after that, satisfactory hemostasis in the surgical field was obtained after a deliberate bleeding control, and the chest was closed 30 min after terminating CPB. Measured ACT values were 160s and 129s respectively after 3 and 6 h of operation (Fig. 1). Aortic cross-clamp time was 90 min, total CPB time was 148 min. Chest tube drainage was 600 ml on the 1st postoperative day and 300 ml on the 2nd day, respectively, and there were no complications in the postoperative period. We ordered acetylsalicylic acid, warfarin, and apixaban the day after the operation. Apixaban was stopped after the required international normalized ratio was reached. The patient was discharged from our hospital on the postoperative 7th day without any notable event. Total transfusions used intraoperatively and in the first 2 days postoperatively were 3 packs of erythrocyte suspension, 4 packs of fresh frozen plasma and 3 packs of platelet suspension.

## Discussion

Unfractionated heparin is the gold standard for anticoagulation during CPB because of its rapid onset, reliable and foreknown effects, and reversibility with protamine sulfate [6]. The major adverse effect of heparin is heparin-induced thrombocytopenia (HIT), a paradox in which an anticoagulant causes a severe procoagulant condition due to antibodies against a complex heparin and platelet factors [7]. Allergic reactions related to heparin are rare [1]. The most common hypersensitivity reaction is urticarial rash. Other symptoms consist of asthma and anaphylaxis [8]. These hypersensitivity reactions have to be distinguished from HIT.

Alternative anticoagulation strategies for CPB are needed in patients who cannot use heparin. Heparin alternatives used for CPB have included non-thrombin inhibitors such as danaparoid, ancrod, and direct thrombin inhibitors (DTI).^9^ Direct thrombin inhibitors are hirudin, argatroban, and bivalirudin. All three DTI drugs bind selectively to circulating and clot-bound thrombin and do not have an antidote. The DTI drugs differ pharmacologically and have been used during CPB.

Hirudin is a natural anti-thrombin and is now produced as recombinant (lepirudin). Several studies and cases are reporting successful use of lepirudin use during CPB. Koster et al. reported that 95% of 57 patients were discharged uneventfully [9]. The main disadvantage of lepirudin is its potential to cause anaphylaxis. There have been seven cases of severe anaphylactic reactions to lepirudin during CPB [10]. In addition to this lepirudin has a relatively long half-life of 60–80 min and is dependent on renal clearance. Patients with renal dysfunction make the halftime unpredictable and may result in excessive bleeding. Also, its monitoring is best achieved by ecarine ACT which is not commercially available everywhere [11].

Argatroban is a synthetic, nonpeptide L-arginine derivative that reversibly inhibits thrombin. It undergoes hepatic elimination with a half-life of 40-50 m but this can be prolonged with hepatic dysfunction. There is very limited data for its use during CPB in the literature. Martin et al. reported six patients with CPB and three had severe coagulopathy that needed threefold use of blood products and clot in the pump was observed in one patient despite elevated ACT [12]. Smith et al. report a case in which argatroban was used for anticoagulation for CPB in a patient allergic to heparin for mitral valve replacement [13].

Bivalirudin is a small peptide that inhibits thrombin reversibly. Bivalirudin is cleared by proteases including thrombin with approximately 80% elimination by enzymatic cleavage and the other 20% is renally eliminated. The elimination half-life of bivalirudin is 20–30 min in the presence of normal renal function. Bivalirudin is currently approved in many countries for patients with unstable angina undergoing percutaneous coronary intervention with provisional use of glycoprotein IIb/IIIa inhibitor and in patients at risk of HIT undergoing percutaneous coronary intervention. According to current American College of Clinical Pharmacology guidelines, it is the preferred agent for patients with HIT requiring urgent cardiac surgery [14]. There are many studies and case reports about the successful use of bivalirudin during CPB. Two and a half times of baseline ACT is generally accepted for initiating CPB while using bivalirudin [15]. ‘EVOLUTION ON ' trial which was a multicentre study that compared bivalirudin anticoagulation with heparin protamine management showed that the safety and efficacy profile was similar between the two groups [16]. In the ‘CHOOSE ON’ trial bivalirudin anticoagulation was used in patients with HIT. The safety and efficacy profiles were similar to before mentioned study [3]. In light of this information we decided to use bivalirudin in our patients. We reviewed the literature and decided to use the dosage suggested in the guideline about anticoagulation during CPB [4]. This dosage regimen consists of a loading dose of 1.0 mg/kg, infusion of 2.5 mg/kg/h, and pump prime of 50 mg. We targeted above 400s ACT for maintaining CPB. It is reasonable to maintain activated clotting time above 480 seconds during CPB. However, values above 400 seconds are frequently considered therapeutic [4].

Given that there is no reversal agent or antidote specifically for bivalirudin, there are some suggested precautions to be taken to maintain a reliable operation while using bivalirudin. In addition, in the study of Koster et al., it is reported that zero-balance modified hemodiafiltration without the addition of vacuum aspiration is effective in improving bivalirudin elimination after CPB [17]. Heparine or heparine related coatings must not be selected. If possible citrate used cell caver can be used in addition to cardiotomy sucker. Cardiotomy sucker should be used frequently and no pooling should be allowed in operation areas. The overriding concern is about the elimination of areas of blood stasis throughout the circuit. Areas of stasis are potentially at risk for clot formation. To prevent clot formation venous reservoir level should be kept as low as possible and the excess volume can be stored in citrate phosphate dextrose bags for later use. Normothermic operation is preferred because elimination times are best known in normothermic situations [5, 18].

In this report, we needed to use more frequent and more amounts of bolus doses to get above 400s ACT levels and reach the safe range with this regimen. We had to increase the infusion rate. Although we initially planned the operation under mild hypothermia, we needed to cool the patient further to reduce bivalirudin elimination. After stopping the infusion, we waited for about 55 min, but ACT levels did not decrease to the desired level and we had to complete the operation while ACT levels were high. This may be the result of both greater hypothermia and the additional use of bivalirudin. ACT level returned to normal after 6 h.

## Conclusion

As a result, our experience with bivalirudin for anticoagulation during CPB in this patient was very convenient. This patient had neither thrombotic nor excessive bleeding complications. However, we can suggest that every patient has to be evaluated individually and dosages can be increased more than planned if needed. Bivalirudin may be suggested as a viable alternative anticoagulant in patients with heparin allergy during CPB.

## Abbreviations

ACT: activated clotting time
CPB: cardiopulmonary bypass
CABG: coronary artery bypass graft
ECT: ecarine clotting time
HIT: heparin-induced thrombocytopenia
DTI: direct thrombin inhibitors
